# Analysis of Cause-of-Death Mortality in Children and Young Adults with Diabetes: A Nationwide 10-Year Follow-Up Cohort Study

**DOI:** 10.3390/children10020358

**Published:** 2023-02-10

**Authors:** Iee-Ho Choi, Sang-Woo Yeom, Sun-Young Kim, Jihye You, Jong-Seung Kim, Minsun Kim

**Affiliations:** 1 Department of Pediatrics, Jeonbuk National University Medical School, Jeonju 54896, Republic of Korea; 2 Department of Otolaryngology-Head and Neck Surgery, Jeonbuk National University Medical School, Jeonju 54896, Republic of Korea; 3 Department of Medical Informatics, Jeonbuk National University Medical School, Jeonju 54896, Republic of Korea; 4 Research Institute of Clinical Medicine of Jeonbuk National University-Biomedical Research Institute of Jeonbuk National University Hospital, Jeonju 54896, Republic of Korea

**Keywords:** diabetes mellitus, death, children, young adults

## Abstract

We examined the associations of clinical characteristics and cause-of-death patterns with mortality in children and young adults (<30 years) with diabetes. We analyzed a nationwide cohort sample from the KNHIS database using propensity score matching from a sample of 1 million people from 2002 to 2013. There were 10,006 individuals in the diabetes mellitus (DM) group and 10,006 in the control (no DM) group. The numbers of deaths were 77 in the DM group and 20 in the control group. The deaths of patients in the DM Group were 3.74 (95% confidence interval (CI) = 2.25–6.21) times higher than in the control group. Type 1 DM, type 2 DM and unspecified DM were 4.52 (95% CI = 1.89–10.82) times, 3.25 (95% CI = 1.95–5.43) times and 10.20 (95% CI = 5.24–20.18) times higher, respectively. Mental disorders were 2.08 times higher in the risk of death (95% CI = 1.27–3.40). Mortality rates have increased in children and young adults with diabetes alone. Therefore, in the future, it is necessary to identify the cause of the increased mortality rate among young diabetic people and select vulnerable groups among them so that early prevention can be achieved.

## 1. Introduction

As the incidence of diabetes continues to increase toward epidemic levels worldwide, better monitoring tools are necessary to identify specific groups of patients with the highest disease complication risk [1]. Although young diabetic patients do not have the common chronic complications associated with diabetic death, fatalities continue to be reported and this needs to be resolved. An understanding of the underlying risks before the Coronavirus disease 2019 (COVID-19) era may be of assistance in solving the problem of young diabetic deaths in the post-COVID-19 era.

Diabetes is a common chronic endocrine disorder in children and adolescents [2]. Diabetes is the seventh leading cause of death in the United States and still an important cause of disability and poor quality of life [3]. Also, diabetes is the sixth leading cause of death in South Korea, with 17.1 deaths per 100,000 population [4]. Diabetes is a risk factor for early death and has been reported as a relatively short-lived condition [5,6,7]. Especially, there are many kinds of research about the risk of death in patients with diabetes. They may vary depending on the presence of common cardiovascular risk factors such as LDL-cholesterol, hypertension and smoking and the severity of the diabetes, such as HbA1c, microalbuminuria and macroalbuminuria [8]. Unlike fixed factors such as gender, age and type of diabetes, factors that can change this risk are actively studied in adults and the elderly. These mainly focus on type 2 diabetes, and there are many attempts to control and prevent it. Therefore, older adults with type 2 diabetes who can manage these risk factors over a long period can reduce the risk of death directly related to cardiovascular complications similar to the general population [8]. However, young diabetics have a relatively high prevalence of type 1 diabetes and a short period of type 2 diabetes, so it is not yet sufficient to grasp and analyze the related situation. In South Korea’s nationwide population-based data, there is a statistically significant increase in age at death in the diabetic population and a significant difference in age at death between the diabetic and non-diabetic populations [5]. However, reports of these reported complications or deaths are mainly focused on adults, and there are few reports of diabetes death at a young age, especially in children and adolescents. 

The aim of this study was to investigate the disparities in diabetes death between adults and children in South Korea. We investigated the changes in mortality among young people with diabetes and compared these trends between those with and without diabetes in the Korean population.

## 2. Materials and Methods

### 2.1. Database and Type of Study

We used data from the National Health Insurance database maintained by the Korean National Health Insurance Service (KNHIS), as well as mortality records and statistics from the National Statistical Information Service (NSIS). The Korea National Health Insurance Service-National Sample Cohort (KNHIS-NSC) consists of approximately 1 million people, 2% of the total 50 million people in Korea. Data on age, sex, residential area and economic status were extracted randomly from the database. In this retrospective cohort study, we analyzed essential clinical factors such as age and sex, and medical-related data such as clinic visit date and diagnosis code.

### 2.2. Diabetes Mellitus (DM) Group

The definition of the experimental cohort was DM for the preceding disease with the diagnostic code E10 (type 1 diabetes), E11 (type 2 diabetes) and E12/13 (unspecified diabetes) (based on the International Classification of Diseases, 10th revision (ICD-10) diagnosis codes) diagnosed between 2002 and 2013. Death was defined as the outcome. There were two inclusion criteria: (1) patients diagnosed with diabetes (type 1, 2 or unspecified) during the period 2002–2013; (2) patients <30 years of age. The commencement date for the DM group was the date of diabetes diagnosis.

### 2.3. Control (Non-DM) Group

From the DM cohort defined above, the control group was calculated by 1:1 propensity score (PS) matching considering nine independent variables (age, sex, economic status, residential area, allergic rhinitis, asthma, atopic dermatitis, cardiac diseases and mental illness) using logistic regression. We performed PS matching using a ‘greedy nearest neighbor’ algorithm with a 1:1 ratio. The confirmation of successful PS matching was determined by the presence or absence of significant imbalances in the figures or the standardized mean difference (SMD) of each group. When the SMD value was less than 0.1, it was judged that the distribution was good with no difference between the two groups [9,10,11,12,13].

### 2.4. Check Points

The patients (DM group) and control group were examined by diagnosis for 12 years from 1 January 2002 to 31 December 2013 to determine whether the primary end point, death, had occurred. The causes of death and accompanying diseases were checked on the death certificates. The types of diabetes were divided by mortality records and the National Health Insurance database, and we included unspecified diabetes.

### 2.5. Statistical Analysis

We divided the subjects between 1 January 2002 and 31 December 2013 into the DM group and the non-DM group and checked the occurrence of death in each group for 12 years thereafter. The independent variables for each group were as follows: 1. Male or female. 2. Old or young separated by diagnosed age of 25 years. 3. Metropolitan, urban or rural. 4. From the 10th quintile for economic status; the upper 50% were defined as high economic status and the rest as low economic status. 5. History/no history of allergic rhinitis, asthma, atopy, cardiac diseases, mental disorders. 6. Accompanied allergic rhinitis (patients whose main illness and sub-illness were ICD-10 with J301, J302, J303, J304), asthma (patients whose main illness and sub-illness were ICD-10 with J45, J46), atopy (patients whose main illness and sub-illness were ICD-10 with L20), cardiac diseases (patients whose main illness and sub-illness ICD-10 started with “I”, “Q2”), mental illness (patients whose main illness and sub-illness ICD-10 started with “F”: mental, behavioral and neurodevelopmental disorders).

Hazard ratios (HRs) and 95% confidence intervals (CIs) for the death of patients with diabetes were estimated using multivariable cox proportional hazards model. All statistical analyses were performed using R version 3.5.3. (R Foundation for Statistical Computing, Vienna, Austria).

### 2.6. Ethics Approval and Consent to Participate

The protocol was approved by the Institutional Review Board (IRB) of Jeonbuk National University Research Council (IRB no. CUH 2021-06-043, date of approval—23 June 2021). The IRB waived the need for informed consent.

## 3. Results

### 3.1. Comparison of Baseline Clinical Characteristics

The baseline clinical characteristics are shown in Table 1. Before PS matching, the DM group and non-DM group were distributed evenly only in four variables (sex, residential area, economic status and allergic rhinitis). However, after PS matching using nine variables (age, sex, economic status, residential area, allergic rhinitis, asthma, atopic dermatitis, cardiac diseases and mental illness), there were 10,006 patients in the DM group and 10,006 in the non-DM group, where there were no statistically significant differences in sex (SMD = 0.003), age (SMD = 0.006), residential area (SMD = 0.003), economic status (SMD = 0.004), allergic rhinitis (SMD = 0.001), asthma (SMD = 0.021), atopy (SMD = 0.013), cardiac illness (SMD = 0.007) and mental illnesses (SMD = 0.003) between the two groups.

### 3.2. Mortality and HR for Death with Diabetes

**Figure 1:**
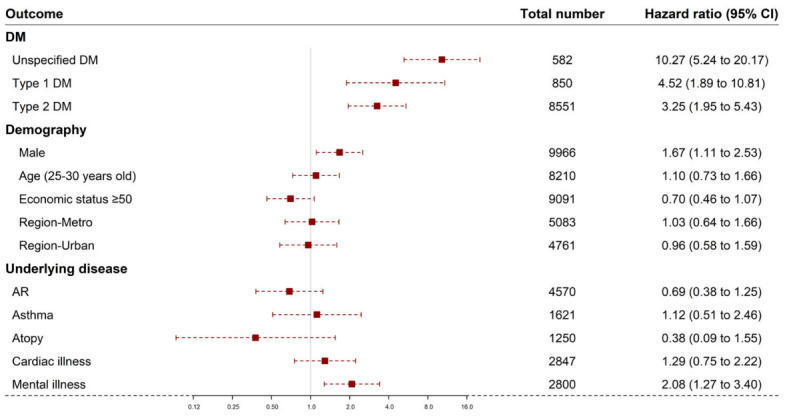
Hazard ratios and 95% confidence intervals for associations of prevalent diabetes and the number of deaths, by selected participant characteristics (DM type 1, 2 or unspecified, male, accompanied mental illness, age (25–30 years), economic status and residential area (metropolitan and urban)). Abbreviations: DM, diabetes mellitus, AR, allergic rhinitis. The red boxes indicate the hazard ratio and the horizontal lines extend from the lower limit to the upper limit of the 95 percent confidence interval.

The mortality rate over the 12-year study period was calculated for each category in the DM and non-DM groups (Table 2 and Figure 1). The number of deaths were 20 and 77 in the non-DM and DM groups, respectively. The HR for death in the type 1 DM group was 4.52 (95% CI: 1.89–10.82); the type 2 DM group was 3.25 (95% CI: 1.95–5.43); unspecified DM was 10.2 (95% CI: 5.24–20.18) compared to the non-DM group. The HR for males was 1.67 (95% CI: 1.11–2.53), compared to females. The HR for those aged ≥25 years was 1.10 (95% CI: 0.73–1.67), compared to those aged <25 years. The HRs for the metropolitan and urban groups were 1.02 (95% CI: 0.64–1.66) and 0.96 (95% CI: 0.58–1.59), respectively, compared to the rural group. The HR for the economic group ≥50th percentile was 0.70 (95% CI: 0.46–1.07), compared to the <50th percentile group. The HRs for the accompanied allergic rhinitis, asthma, atopy and cardiac disease groups were all insignificant at 0.68 (95% CI: 0.38–1.25), 1.11 (95% CI: 0.51–2.45), 0.37 (95% CI: 0.09–1.55) and 1.29 (95% CI: 0.75–2.22) compared to the group with no corresponding diseases. The HR for the accompanied mental illness group was 2.08 (95% CI: 1.27–3.40), compared to the group with no accompanied mental illness.

### 3.3. The Number of Patients and Deaths According to the Follow-Up Date

**Figure 2:**
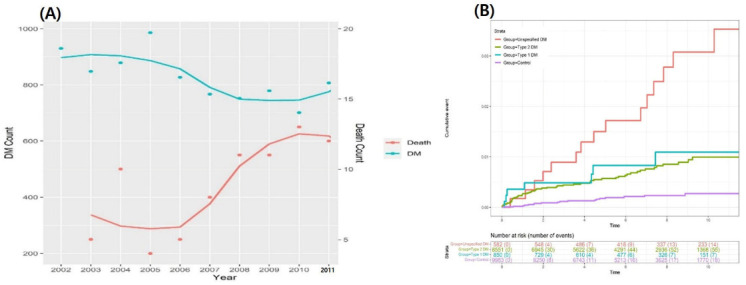
The number of diagnosed patients and deaths with diabetes mellitus (**A**). Cumulative hazard plot for death between the DM type and the control group (**B**). Abbreviations: DM, diabetes mellitus.

The incidence curve for death in the DM group is shown in Figure 2A. The number of deaths in diabetic patients continues to increase annually. The cumulative hazard plot for death in this study is shown in Figure 2B. A progressive increase in mortality was statistically significantly associated with diabetes throughout this study period.

### 3.4. Hazard Ratios for Death during the 12 yr Study Period

**Figure 3:**
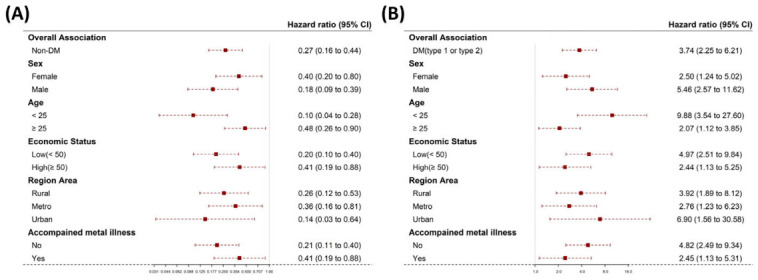
Hazard ratios and 95% confidence intervals of mortality according to the selected participant characteristics by (sex, age, economic status and accompanied mental illness) in non-diabetes (**A**) and diabetes (type 1 or 2) (**B**). Abbreviations: DM, diabetes mellitus. The red boxes indicate the hazard ratio and the horizontal lines extend from the lower limit to the upper limit of the 95 percent confidence interval.

**Figure 4:**
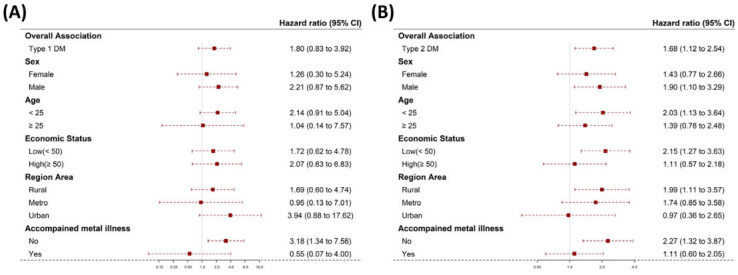
Hazard ratios and 95% confidence intervals of mortality according to the selected participant characteristics by (sex, age, economic status and accompanied mental illness) in diabetes type 1 (**A**) and 2 (**B**). Abbreviations: DM, diabetes mellitus. The red boxes indicate the hazard ratio and the horizontal lines extend from the lower limit to the upper limit of the 95 percent confidence interval.

Type 1 or type 2 diabetes patients had higher HRs for death than the non-DM group. In the non-DM group, contrary to the DM group results, those aged 25–30 years had a trend of higher HRs for death than the others, despite no statistical significance (Figure 3A). In the DM (type 1 or type 2 DM) group, there was no statistical evidence of a link in mortality between diabetes and sex, age, economic status or accompanied mental illness (Figure 3B). Despite the higher HR in type 1 DM compared to type 2 DM, there was no significant difference between them. There was no statistical evidence of a link in mortality between DM types and sex, age, economic status or accompanied mental illness (Figure 4A,B).

## 4. Discussion

Our study highlights the related factors for death in young diabetic patients who were diagnosed before the age of 30. These data may be important for health service planning. The population aged <25 years in South Korea was continuously decreasing from 2002 to 2013, the period of this study [14]. However, during this period the number of deaths among people with diabetes at such a young age increased. Because children and young adults have significantly lower chronic disease risks, such as hypertension, chronic kidney diseases, hyperlipidemia, etc., than adults [15], we hypothesized that young diabetes patient death would have a specific social condition. Therefore, we analyzed the hazard ratio and mortality by diabetes type, sex, age, economic status, etc. In our study, diabetes is strongly related to the likelihood of death. Although there was no significance, the high mortality was in the age < 25 years in diabetic people. This pattern was contrary in the non-diabetic group. It may reflect that young patients with diabetes have a higher risk of death, so it is vital to find the possible death-related factors and make improvements.

The mortality rate of young diabetic patients is higher than that of the general non-diabetic population, even after accounting for causes. These results showed a similar pattern in adult studies [16,17,18]. In particular, the mortality rate of adult patients with diabetes accompanied by cardiovascular system issues was high [19,20,21,22,23]. Furthermore, Gu et al., reported that the disparity in ischemic heart disease mortality between people with and without diabetes continued to increase [24]. It is known that the effects of diabetes on the mortality rate of respiratory and kidney diseases are also diverse [25]. In a Danish nationwide cohort study [26] of children and adolescents under 50 years of age and young adults with diabetes, which collected the cause of death from the death certificate and autopsy record, the risk of cardiovascular death and lung disease contributed to the increased mortality rate powerfully. The following causes of death in diabetic patients were endocrine diseases and cancer. Considering that cancer and renal failure accounted for a high rate in previous studies of elderly adults with diabetes, there were differences in the comorbidities of mortality by age group. In addition, type 1 diabetic patients (age 1–49 years) had a seven-fold higher mortality rate than the general population, and cardiovascular mortality was heightened by eleven times. Adult males aged 21–30 with type 2 diabetes had a significantly higher mortality rate than non-diabetic males. In addition, the mortality rate was 2–4 times higher in middle-aged patients with type 2 diabetes. A recent study [27] reported that the age at diagnosis of type 1 diabetes is an essential factor in mortality due to cardiovascular disease. Those diagnosed with type 1 diabetes before age 10 had an up to five-fold higher risk of developing cardiovascular disease than those diagnosed between the ages of 26 and 30. Therefore, it was announced that cardiovascular monitoring was vital to prevent death in young diabetic patients. Furthermore, there are attempts to analyze and overcome the impact through various analyses and research methods at the regional or national level. In particular, children, adolescents and young adults have different health conditions than the elderly, so different approaches and analyses than those related to adults are needed.

The two primary forms of diabetes are type 1 and type 2 diabetes. Both forms can be diagnosed at any age, but type 1 diabetes is higher in childhood than in adults. The age of diagnosis of type 1 diabetes is highest at about 5 to 6 years of age and occurs in many cases at 11 to 13 years of age [28]. Although type 1 diabetes is the most common form of diabetes among young people, type 2 diabetes is becoming an increasingly crucial public health problem worldwide [8]. The cause of diabetes still needs much research and is known to be complex, including immune system disturbances, genetics, environmental factors, etc., but the fundamental causes can be classified as follows; decreased insulin secretion from the pancreas (type 1 diabetes), resistance to insulin action and the imbalance of the insulin secretion response (type 2 diabetes) [29].

While the incidence of type 1 diabetes in young people is relatively stable, the prevalence of type 2 diabetes is increasing rapidly. Type 2 diabetes used to be called “adult-onset” diabetes because it was rarely diagnosed in children [28]. However, as childhood obesity rates increase, this disease diagnosis rate in more and more children is rising, and the age of diagnosis is decreasing [28,29]. It is known that various factors, such as genetics, physiology and lifestyles, influence the occurrence of type 2 diabetes [29]. The continued exposure to these conditions from childhood means that more and more of the population will live with diabetes from a young age. Therefore, the disease and related complications can be a factor that increases the socio-economic burden on the individual and society. In this study, diabetes has a high correlation with mortality, and the same result was shown even when considering common diseases at a young age. This circumstance is why individuals and society should pay attention to these changes and prepare countermeasures quickly and concretely. In addition, given the much higher mortality rate of unspecified diabetes than type 1 or type 2 diabetes, it would be meaningful to analyze these unspecified types and find related factors in the future. Other risk factors contributing to type 2 diabetes in children include a family history of diabetes, being born to a mother with diabetes during pregnancy (gestational diabetes), or other medical problems that affect insulin metabolism [28].

Diabetic death in children, adolescents and young adults may result from the acute rather than chronic complications of diabetes [30]. According to US reports, diabetes is one of the most common childhood chronic diseases, reporting 283,000 children and adolescents with diabetes in America [31]. The acute complications of diabetes, such as hypoglycemia and diabetic ketoacidosis, increase the risk of death in children and adolescents with diabetes [2]. In 2012, The Centers for Disease Control and Prevention (CDC) reported a 61% reduction in diabetes mortality among Americans aged <19 years between 1968 and 2009 [2]. The CDC analyzed data from the National Vital Statistics System on deaths between the ages of 1–19 years in the United States for the period 2000–2014, where diabetes was listed as a leading cause of death; there were racial/ethnic differences; in particular, black children and adolescents reported the highest mortality rates [2]. In order to reduce deaths from diabetes in children and adolescents, measures such as the rapid recognition of diabetes symptoms (including hypoglycemic symptoms), rapid access to diabetes treatment and education and the preventive management of diabetic ketoacidosis such as the use of continuous glucose monitoring should be implemented. Continued understanding and action are needed to reduce diabetes mortality among young people, including children.

Generally, research involving health, mortality and socioeconomic information is conducted with well-informed target populations [32]. Diabetes is a representative disease for which such information is continuously increasing. It tracked and tried to improve the process of reducing socioeconomic disparities to improve diabetes-related mortality, especially in various areas. However, these studies have focused on adults. Saydah et al., explained lower education levels and lower family income as typical factors that increase mortality in adults with diabetes [32]. Studies in adults with type 1 diabetes [33] and type 2 diabetes [34] also reported that low socioeconomic status and the presence or not of chronic complications were associated with mortality. Pediatric and young diabetes patients have a different environment than adult patients, and the significance is that they need longer-term national measures than adults. Although the diabetes group did not have statistical significance in this study, the mortality risk was high in young age and low economic status. It showed the necessity for further follow-up studies related to this.

Our study also showed that the mental health of young diabetic patients is very important. When analyzed alongside common diseases that can accompany children/adolescents and young adults, mental illness was found to be associated with mortality risk. In a recent study [35], it was reported that diabetes itself is associated with increased morbidity, mortality and decreased quality of life, which is related to several psychological negatives. The importance of finding and applying an appropriate management method to reduce the psychosocial burden was also argued to overcome this. This also confirms the necessity for our study, and it is another task to develop appropriate care methods for diabetic patients with increased mental burden in the COVID-19 era [36]. It is not that diabetic people have more issues, such as social problems, than non-diabetic people. Nevertheless, in the Diabetes UK cohort study of type 1 diabetics who died at an age of less than 40, it was reported that acute death and psychosocial and socioeconomic factors might be more related than death due to long-term complications [37]. They introduced that many deaths of diabetic patients after 30 are connected to chronic complications of diabetes. Still, many acute diabetes-related events occur in those under 30 years of age. Furthermore, societal factors are associated with diabetes management. When diabetes and socioeconomic problems are mixed, it leads to reduced treatment adherence and reduces control, which can be related to sudden death [38]. Risk factors for premature death included living alone, drug abuse and past psychiatric problems [37].

We investigated the mortality rates of diabetes in those aged <30 years from 2002–2013 using death certificate information. Death was defined as the International Classification of Diseases (ICD), 10th edition, with default cause codes of E10–E13. Therefore, our study had some limitations. First, a more detailed analysis was not possible due to the small number of deaths due to diabetes, which made it difficult to distinguish subtle trend changes. Second, by using the ICD codes, it was not possible to analyze the death data by distinguishing between the E12 and E13 codes, so they were separated into different groups and compared. Third, the analyses of individual healthcare status, diabetes self-management and the education status of patients themselves and their guardians, and differences in access to diabetes management were not conducted together. We could not collect these data because this information is not included in the KNHIS reports. Fourth, we compared socioeconomic status using the mortality data of diabetic patients. It did not collect comorbidities. Large-scale studies and follow-up studies on comorbidities related to mortality are needed.

To the best of our knowledge, this is the first study to analyze diabetes mortality and related factors among young people aged <30 years using South Korean national data in the pre-COVID era. The mortality rate of young diabetic patients is continuously increasing, which indicates that further studies and investigations are necessary.

## 5. Conclusions

The prevalence of death in patients with diabetes is 3.74 times higher than in patients without diabetes. Diabetes is the most mortality-increasing factor by itself in young diabetes patients. This phenomenon is not influenced by sex, age, economic status, or mental disorders. Clinicians should pay close attention to the care of young diabetes patients. Future studies should further investigate the causes of death in young diabetic patients because it is important to find preventive measures.

## Figures and Tables

**Table 1:** Characteristics of the control (non-DM) and study (DM) groups.

Variables	Before Matched			After Matched		
	Non-DM Group (*n* = 374,601)	DM Group (*n* = 10,006)	SMD	Non-DM Group (*n* = 10,006)	DM Group (*n* = 10,006)	SMD
Sex			0.039			0.003
Male	194,754	5007		4992	5007	
Female	179,847	4999		5014	4999	
Age			0.447			0.006
<25	296,655	5909		5877	5909	
≥25	77,946	4097		4129	4097	
Residential area			0.088			0.003
Rural	199,135	5077		5075	5077	
Metro	100,060	2552		2541	2552	
Urban	75,406	2377		2390	2377	
Economic status			0.077			0.004
≧50 (high)	184,614	4545		4567	4545	
<50 (low)	189,987	5461		5439	5461	
Allergic rhinnitis			0.009			0.001
No	287,502	7716		7719	7716	
Yes	87,099	2290		2287	2290	
Asthma			0.152			0.021
No	325,550	9166		9223	9166	
Yes	49,051	840		783	840	
Atopy			0.135			0.013
No	336,683	9365		9397	9365	
Yes	37,918	641		609	641	
Cardiac illness			0.345			0.007
No	361,965	8746		8770	8746	
Yes	12,636	1260		1236	1260	
Mental illness			0.302			0.003
No	354,919	8597		8607	8597	
Yes	19,682	1409		1399	1409	
Death			0.116			0.101
No	374,133	9907		9985	9907	
Yes	468	99		21	99	

Abbreviations: DM, diabetes mellitus.

**Table 2:** Mortality and hazard ratios for death during the 12 yr study period.

Group	Total	Number of Mortality Cases (%)	Hazard Ratio (95% Confidence Interval, Lower–Upper)
Control	9983	20 (0.2)	1
Type 1 DM	850	7 (0.82)	4.52 (1.89–10.82)
Type 2 DM	8551	55 (0.64)	3.25 (1.95–5.43)
Unspecified DM	582	15 (2.58)	10.20 (5.24–20.18)
Sex			
Female	10,000	37 (0.37)	1
Male	9966	60 (0.6)	1.67 (1.11–2.53)
Age			
< 25	11,756	52 (0.44)	1
≧ 25	8210	45 (0.55)	1.10 (0.73–1.67)
Residential Area			
Rural	10,122	49 (0.48)	1
Urban	4761	22 (0.46)	0.96 (0.58–1.59)
Metropolitan	5083	26 (0.51)	1.02 (0.64–1.66)
Economic Status			
<50 (low)	10,875	61 (0.56)	1
≧50 (high)	9091	36 (0.4)	0.70 (0.46–1.07)
Underlying disease			
Allergic rhinnitis			
No	15,396	84 (0.55)	1
Yes	4570	13 (0.28)	0.68 (0.38–1.25)
Asthma			
No	18,345	90 (0.49)	1
Yes	1621	7 (0.43)	1.11(0.51–2.45)
Atopy			
No	18,716	95 (0.51)	1
Yes	1250	2 (0.16)	0.37(0.09–1.55)
Cardiac diseases			
No	17,479	81 (0.46)	1
Yes	2487	16 (0.64)	1.29(0.75–2.22)
Mental Disorder			
No	17,166	76 (0.44)	1
Yes	2800	21 (0.75)	2.08 (1.27–3.40)

Abbreviations: DM, diabetes mellitus.

## Data Availability

The data used in this study are available from the corresponding authors upon request.
